# Fine Particulate Air Pollution and Hospital Utilization for Upper Respiratory Tract Infections in Beijing, China

**DOI:** 10.3390/ijerph16040533

**Published:** 2019-02-13

**Authors:** Daitao Zhang, Yaohua Tian, Yi Zhang, Yaying Cao, Quanyi Wang, Yonghua Hu

**Affiliations:** 1 Department of Epidemiology and Biostatistics, School of Public Health, Peking University, Beijing 100191, China; zdt016@163.com (D.Z.); yaohua_tian@bjmu.edu.cn (Y.T.); caoyaying@bjmu.edu.cn (Y.C.); 2 Institute of Infectious Diseases and Endemic Diseases Control, Beijing Municipal Center for Disease Prevention and Control & Beijing Research Center for Preventive Medicine, Beijing 100013, China; zps347@163.com (Y.Z.); bjcdcxm@126.com (Q.W.)

**Keywords:** fine particulate matter, PM_2.5_, upper respiratory tract infections, generalized additive model, time series

## Abstract

Few studies have examined the association between fine particulate matter (PM_2.5_) and upper respiratory tract infections (URTI) in urban cities. The principal aim of the present study was to evaluate the short-term impact of PM_2.5_ on the incidence of URTI in Beijing, China. Data on hospital visits due to URTI from 1 October 2010 to 30 September 2012 were obtained from the Beijing Medical Claim Data for Employees, a health insurance database. Daily PM_2.5_ concentration was acquired from the embassy of the United States of America (US) located in Beijing. A generalized additive Poisson model was used to analyze the effect of PM_2.5_ on hospital visits for URTI. We found that a 10 μg/m^3^ increase in PM_2.5_ concentration was associated with 0.84% (95% CI, 0.05–1.64%) increase in hospital admissions for URTI at lag 0–3 days, but there were no significant associations with emergency room or outpatient visits. Compared to females, males were more likely to be hospitalized for URTI when the PM_2.5_ level increased, but other findings did not differ by age group or gender. The study suggests that short-term variations in PM_2.5_ concentrations have small but detectable impacts on hospital utilization due to URTI in adults.

## 1. Introduction

Fine particulate matter (of aerodynamic diameter ≤ 2.5 µm) (PM_2.5_) is an important cause of mortality and morbidity worldwide [1,2]. Numerous studies have shown a strong association between PM_2.5_ and respiratory health [3,4]. Upper respiratory tract infections (URTI) are infections of the mouth, nose, throat, larynx (voice box), and trachea (windpipe). They are the main causes of medical consultation [5] and the leading causes of misuse of antibiotics worldwide [6]. Globally, at least 10–25% of visits to general practitioners and primary care physicians every year are related to URTI [7], with an estimated two to four episodes per person per year in adults [8].

In mainland China, a number of studies have reported a positive relationship between PM_2.5_ and health in recent years [9,10,11]. Studies conducted in other countries and regions have also demonstrated a positive relationship between PM_2.5_ and URTI [12,13,14,15]. For instance, short-term exposure to PM_2.5_ has been shown to be related to lung dysfunction or respiratory diseases [16], while higher PM_2.5_ concentrations have been associated with an increase in emergency department and outpatient visits for URTI in children [9,12,17].

The majority of previous studies were focused on exploring the association between PM_2.5_ and URTI among children, who are more susceptible to URTI due to their immature respiratory systems and exposure-relevant behaviors [11,12,17]. However, few studies have qualified the impact of PM_2.5_ on adults. In our study, we aimed to explore the effects of PM_2.5_ on hospital utilization for URTI in adults using the data from all employees and retired residents who have medical insurance in Beijing.

## 2. Material and Methods

### 2.1. Background of Study Area

Beijing is located in northern China and experiences a temperate semihumid continental monsoon climate. It has a land area of 164,100 km^2^ and is the second largest city in China, with a permanent resident population of more than 20 million. According to the public report of social security in Beijing, about 14.7 million (68.5%) of Beijing’s permanent resident population possess basic medical insurance for working or retired employees, and a total of 1691 hospitals as well as primary medical and health service institutions are covered by this basic medical insurance.

Air pollution varies from city to city depending on factors such as the sources of pollutants, geography, climate, and the type and size of particles [18,19]. It has been reported that the main components of PM_2.5_ in Beijing are higher secondary inorganic aerosols, organic carbon, soil dust, and typical elements (Si, Ca, Fe, Mn, Cu, Pb, and K), and they are generated from secondary aerosols, coal combustion, biomass/waste burning, traffic-related pollution, long-range transport, and fugitive soil and sand dust [20,21].

### 2.2. Outcome Measures

We obtained reports of primary discharge diagnosis (using the International Classification of Diseases, 10th Revision (ICD-10) codes J45 and J46) from the Beijing Medical Claim Data for Employees from 1 October 2010 to 30 September 2012. The data was from the system of basic medical insurance for working or retired employees in Beijing. In many countries, the increase in hospital visits related to the increase in PM_2.5_ may not appear timely for the reason that making appointments are the main means for patients to visit doctors [22]. As a result, it is difficult to demonstrate the relationship between PM_2.5_ and daily total hospital utilization. However, in China, outpatient visits are generally unscheduled and follow the first-come, first-served rule [23]. Most people who are sick can go to hospitals immediately. Hence, the hospital visiting records in China can supply more reliable information on the morbidity of URTI. The data included in our study were classified into hospital (i) emergency department visits, (ii) admissions, and (iii) outpatient visits for URTI.

### 2.3. PM_2.5_ and Weather Data

We obtained publicly available contemporaneous hourly concentrations of PM_2.5_ collected from the air quality monitoring station located at the US embassy in Chaoyang district, Beijing. Previous studies have reported that ambient air quality data can be used as a proxy for the personal exposure among individuals residing <40 km from the monitoring station [24,25]. In Beijing, 79.2% of inhabitants live within this area [25].

We obtained contemporaneous daily mean ambient temperature and daily mean relative humidity for Beijing from the Chinese Meteorological Bureau. As previous studies have shown that temperature and humidity are potential confounders of the association between ambient air pollution and diseases [26,27], we included them in our models to obtain more accurate estimates of the independent effects of PM_2.5_ on hospital utilization for URTI.

### 2.4. Statistical Analysis

We used an ecological time-series study design to analyze the short-term associations between PM_2.5_ and (i) emergency department visits, (ii) admissions, and (iii) outpatient visits for URTI. We obtained daily mean concentrations of PM_2.5_ by taking the mean hourly concentrations for each day. Smoothing function was applied to analyze the exposure–response relationship between the log-relative URTI morbidity risk and the PM_2.5_ level. We used a generalized additive model (GAM) assuming a quasi-Poisson distribution and allowing for overdispersion. We analyzed the data on a daily timescale and accounted for the long-term trend, seasonality, day of the week, public holidays, and the potential confounding effects of mean ambient temperature and mean relative humidity (see Equation (1) below). The percentage changes and 95% confidence intervals (CIs) in daily URTI hospital utilization related to per 10 μg/m^3^ increase in PM_2.5_ concentrations were calculated. We obtained gender- and age group-specific estimates in separate regression models.

(1) $$\log\left[E\left(Y_{t}\right)\right]=\alpha +\beta \mathrm{PM}2.5+\gamma \mathrm{day}\;\mathrm{of}\;\mathrm{week}+\delta \mathrm{public}\;\mathrm{holiday}+\mathrm{ps}\left(\mathrm{calender}\;\mathrm{time},14\right)\;+\mathrm{ps}\left(\mathrm{temperature},3\right)+\mathrm{ps}\left(\mathrm{relative}\;\mathrm{humidity},3\right)$$

where $E\left(Y_{t}\right)$ represents the expected counts of each outcome measure at day t; β denotes the log-relative URTI morbidity related to every 10 μg/m^3^ increase in PM_2.5_; γ is the coefficient of the day of week; δ is the coefficient of public holiday, which was controlled as a binary variable; ps () represents penalized spline function. A natural spline of calendar day with 14 degrees of freedom (*df*) was used to adjust for the unmeasured time trends in URTI during the study period (about 24 months). A natural spline of daily mean temperature with 3 *df* and a natural spline of daily mean relative humidity with 3 *df* was used to adjust for the potential confounding effects of weather conditions [27]. We represented the effect of a 10-unit increase in the mean daily concentration of PM_2.5_ on the outcome measures using (incidence rate ratio (IRR) − 1) × 100.

In sensitivity analysis, we tested a range of *df* values to examine if the initial choice of how calendar time, temperature, and relative humidity was modeled would influence the effect estimates for PM_2.5_. For calendar day, we used *df* values of 10, 12, 16, and 18; for temperature and humidity, we used *df* values of 2, 4, 5, and 6.

Because previous studies have shown little evidence of a strong association between PM_2.5_ level and adverse health effects beyond three days, we used a distributed lag model to estimate the immediate and lagged effects of PM_2.5_ by fitting terms to represent 0, 1, 2, 3 day(s) and 0–3 average lagged days of PM_2.5_ concentration [27]. Stratified models by age or gender were fitted, and Z-test was performed to examine the statistical significance of different subgroups. We assessed statistical significance at the 5% level and indicated the 95% confidence intervals (95% CI) and *p*-values for each effect estimate. All the analyses were undertaken in *R* (V.3.2.2, R Development Core Team).

## 3. Results

Over the study duration, there were 533,876 hospital emergency department visits, 4276 hospital admissions, and 16,750,514 outpatient visits for URTI. The proportions of total hospital utilization, outpatient visits, and emergency room visits of males were all less than those of females. However, the percentage of males hospitalized for URTI was a bit more than that of females. The reports on patients aged 65 and above for all kinds of hospital utilizations were much less than those of younger adults (Table 1).

A daily average of 23,651 (12,386) total hospital utilization, 22,915 (12,345) outpatient visits, 730 (325) emergency department visits, and 7 (4) hospital admissions relating to URTI were found in the study period. The daily mean PM_2.5_ concentration over the study duration was 96.7 µg/m^3^. There were 369 (50.5%) days when the daily PM_2.5_ levels exceeded the daily mean PM_2.5_ concentrations of the Chinese Ambient Air Quality Standards Grade II (75 μg/m^3^). There were only 113 (15.6%) days where the PM_2.5_ concentrations met the WHO air quality standards for daily mean PM_2.5_ concentrations (25 μg/m^3^). The mean daily temperature and relative humidity were 13.3 °C and 49.3%, respectively (Table 2).

Spearman’s correlation tests were conducted to test the correlation of the lagged PM_2.5_ data. The results showed that the correlation between lag 0 and lag 1, lag 0 and lag 2, and lag 0 and lag 3 were 0.532 (*p* < 0.001), 0.197 (*p* < 0.001), and 0.045 (*p* = 0.220), respectively.

After adjusting for calendar time, day of the week, public holiday, and weather conditions, no significant associations were found between the increase in PM_2.5_ concentrations and total hospital utilization, outpatient visits, and emergency room visits. However, a delayed increase in hospital admissions for URTI was demonstrated to follow the rise in PM_2.5_ concentrations. The exposure–response curve of fine particulate matter (PM_2.5_) concentrations at lag 3 days and hospital admissions for URTI was showed in Figure S1. A 10 μg/m^3^ increase in PM_2.5_ concentration was associated with a 0.53% (95% CI, 0.01–1.05%) delayed increase in admissions two days later and a 0.59% (95% CI, 0.06–1.11%) delayed increase in hospital admissions three days later. Additionally, a 0.84% (95% CI, 0.05–1.64%) increase in hospital admissions was found related to the four-day moving average (from current day to the previous three days) of PM_2.5_ (Table 3). However, there were no associations between PM_2.5_ concentrations and any of the outcome measures on the same day. As for the outpatient visits, its largest magnitude association with PM_2.5_ was observed at lag 0, with a 10 μg/m^3^ increase in lag 0 PM_2.5_ being associated with a 0.31% (95% CI: −0.07, 0.69) increase in URTI-related outpatient visits; however, this association was not statistically significant (*p* = 0.113).

As shown in Table 4, the impact of a 10 μg/m^3^ increase in lag 3 PM_2.5_ on hospital admissions was considerably greater in males than in females (0.90% versus 0.45%, interaction *p*-Value < 0.001). No significant interactions were detected by sex or age for total outpatient or emergency room visits or by age for hospital admissions.

The estimated effect of PM_2.5_ on the outcome measures did not differ greatly after varying the number of *df* used for the spline functions for time, mean ambient air temperature, and mean relative humidity (see Supplementary Information). This suggests that the initial choice of *dfs* did not alter the estimated relationship between PM_2.5_ and URTI morbidity to an important degree (see Table S1 in Supplementary Information).

## 4. Discussion

In this study, we sought to examine the relationship between short-term variations in PM_2.5_ and hospital utilization for URTI. We found a positive and delayed relationship between daily PM_2.5_ concentrations and hospital admissions for URTI. No evidence of associations between PM_2.5_ and total hospital utilization, outpatient visits, or emergency room visits was observed.

Previous studies have reported a positive relationship between PM_2.5_ and hospital admissions for URTI [28,29]. The lack of an observable immediate relationship between PM_2.5_ and hospital utilization for URTI is not surprising. In Beijing, it is not unusual to experience administrative delays between the outpatient diagnosis for serious URTI and hospital admissions due to the time taken to assess the severity of the disease and to assign hospitalization space. No significant associations between the increase in PM_2.5_ level and total hospital utilization, outpatient visits, and emergency room visits were found in our study, which was different from some previous studies [9,12,17]. We offer two explanations for these results. Firstly, the majority of subjects in previous studies were children, whereas the subjects involved in our study were adults. Unlike children, adults with URTI may choose to purchase medicines directly from pharmacies instead of visiting hospitals, which might have lowered the ability of our study to detect the increasing number of individuals affected by PM_2.5_. Further studies involving more data, such as the consumption of URTI-related medicines from pharmacies, are needed to explore the association between PM_2.5_ and outpatient and emergency room visits for URTI. Secondly, it was not until 1 March 2012 that the PM_2.5_ level was required to be included in the air quality standards in China [30]. Before that, the ambient air pollution in China had dramatically worsened due to the rapid increase in industrialization, automobile use, and energy consumption since 2000 [31]. The average concentration of PM_2.5_ in our study was much higher than those in some western countries [31,32,33]. Hence, the relationship between high PM_2.5_ level and outpatient and emergency room visits might not be detected in high concentrations of ambient PM_2.5_. In this study, the percentage changes of hospital admissions for URTI related to PM_2.5_ were significantly higher for males than for females. This is different from the results of some previous studies, where females have been found to be usually at higher risk of respiratory diseases after PM_2.5_ exposure than males [10,34]. However, the subjects of these studies included both children and adults, and age-specific results were not available. In this study, the older population was found to be more likely to be hospitalized for URTI after short-term exposure to PM_2.5_. This is consistent with other previous studies. The reason for this might be the weaker immune systems and higher prevalence of chronic respiratory diseases in the elderly population [10].

There are several limitations in our study. Firstly, PM_2.5_ was the only air pollutant examined, and therefore we were unable to adjust for the potential confounding effects of other air pollutants. Secondly, the PM_2.5_ data used in our study was obtained from a stationary monitoring site. This might have underestimated the effect of PM_2.5_ [35], and we were unable to analyze the effects of spatial variations on the association between PM_2.5_ and URTI. Thirdly, the data was from the Beijing Medical Claim Data for Employees, which only covers employed or retired adults. These subjects would be healthier than the general population, including those unable to work due to ill health. This might have potentially underestimated the negative health impact of increased PM_2.5_ on adults with URTI.

## 5. Conclusions

Our study found a positive and delayed association between daily PM_2.5_ concentrations and hospital admissions for URTI in adults. Compared to females, males were more likely to be hospitalized for URTI when the PM_2.5_ level increased. Our study findings may be useful for hospitals seeking to improve resource allocation plans related to variations in PM_2.5_ concentrations.

## Figures and Tables

**Table 1:** Demographic feature of upper respiratory tract infections (URTI)-related hospital utilization from 1 October 2010 to 30 September 2012 in Beijing, China.

Variables	Total Hospital Utilization *n* = 17,288,666	Outpatient Visits *n* = 16,750,514	Emergency Room Visits *n* = 533,876	Hospital Admissions *n* = 4276
Male gender, *n* (%)	6,721,181 (38.9)	6,494,257 (38.8)	224,700 (42.1)	2224 (52.0)
Age ≥ 65, *n* (%)	5,562,495 (32.2)	5,491,425 (32.8)	70,124 (13.1)	946 (22.1)

**Table 2:** Statistical summary of the number of daily hospital visits for URTI, daily fine particulate matter (PM_2.5_) concentrations, and weather conditions from 10 October 2010 to 30 September 2012 in Beijing, China.

Variable	Mean ± SD	Minimum	Percentile			Maximum
			25th	50th	75th	
Daily hospital visits	23,651 ± 12,386	2191	15,381	20,425	32,307	91,579
Outpatient visits	22,915 ± 12,345	1494	14,738	19,909	31,498	90,700
Emergency room visits	730 ± 325	180	471	700	951	1959
Hospital admissions	7 ± 4	1	3	6	9	27
PM_2.5_ (μg/m^3^)	96.7 ± 78.4	2.9	36.9	77.2	127.5	492.8
Temperature (°C)	13.3 ± 11.3	−7.8	2.1	15.6	23.8	31.3
Relative humidity (%)	49.3 ± 21.2	9	29	49	67	97

**Table 3:** The effect of a 10 μg/m^3^ increase in fine particulate matter (PM_2.5_) concentration on URTI-related hospital utilizations for different lag structures.

Hospital Service	Lag Days	Percentage Change	95% CI	*p*
Total hospital utilization	Lag 0 days	0.30	−0.06, 0.66	0.104
	Lag 1 days	−0.09	−0.41, 0.22	0.565
	Lag 2 days	−0.02	−0.30, 0.25	0.863
	Lag 3 days	0.01	−0.26, 0.28	0.919
	Lag 0–3 days	0.06	−0.38, 0.49	0.794
Outpatient visits	Lag 0 days	0.31	−0.07, 0.69	0.113
	Lag 1 days	−0.09	−0.43, 0.24	0.582
	Lag 2 days	−0.02	−0.31, 0.27	0.890
	Lag 3 days	0.01	−0.27, 0.30	0.918
	Lag 0–3 days	0.06	−0.39, 0.52	0.785
Emergency room visits	Lag 0 days	0.16	−0.14, 0.47	0.293
	Lag 1 days	−0.15	−0.42, 0.13	0.299
	Lag 2 days	−0.19	−0.43, 0.05	0.127
	Lag 3 days	−0.16	−0.39, 0.07	0.166
	Lag 0–3 days	−0.23	−0.61, 0.14	0.226
Hospital admissions	Lag 0 days	0.00	−0.68, 0.69	0.996
	Lag 1 days	0.42	−0.17, 1.00	0.163
	Lag 2 days	0.53	0.01, 1.05	0.047
	Lag 3 days	0.59	0.06, 1.11	0.029
	Lag 0–3 days	0.84	0.05, 1.64	0.039

**Table 4:** The effect of a 10 μg/m^3^ increase in fine particulate matter (PM_2.5_) concentration on URTI-related hospital utilizations by sex and age.

Hospital Service	Percentage Change	95% CI	*p*−Value
Total hospital utilization ^a^			
Sex			0.912
Male	0.28	−0.05, 0.61	
Female	0.31	−0.07, 0.69	
Age (year)			0.622
<65	0.26	−0.08, 0.60	
≥65	0.39	0.01, 0.79	
Outpatient visits ^a^			
Sex			0.916
Male	0.29	−0.06, 0.64	
Female	0.32	−0.08, 0.72	
Age (year)			0.645
<65	0.27	−0.09, 0.63	
≥65	0.40	−0.03, 0.82	
Emergency department visits ^a^			
Sex			0.964
Male	0.16	−0.14, 0.46	
Female	0.17	−0.15, 0.48	
Age (year)			0.822
<65	0.15	−0.14, 0.44	
≥65	0.21	−0.22, 0.64	
Hospital admissions ^b^			
Sex			<0.001
Male	0.90	0.24, 1.56	
Female	0.45	−0.28, 1.17	
Age (year)			0.665
<65	0.62	−0.01, 1.25	
≥65	0.87	−0.04, 1.78	

^a^ Lag 0 concentrations were used for total hospital utilization, outpatient visits, and emergency department visits. ^b^ lag 3 concentrations were used for hospital admissions.

## Supplementary Materials

The following are available online at http://www.mdpi.com/1660-4601/16/4/533/s1, Figure S1: The exposure–response curve of fine particulate matter (PM_2.5_) concentrations at lag 3 days and hospital admissions for URTI between 1 October 2010 and 30 September 2012 in Beijing, China. Note: The *x*-axis is the PM_2.5_ concentrations (mg/m^3^) at lag 3 days. The *y*-axis is the predicted log (relative risk (RR)). The curve, after adjusting for temperature, relative humidity, day of week, public holiday, and calendar time, is shown by the solid line, and the dotted lines represent the 95% CI. Table S1: Percentage change with 95% CI in all three kinds of hospital utilizations for URTI related to a 10 μg/m^3^ increase in fine particulate matter (PM_2.5_) level at lag 3 days, by different degree of freedom (*df*) for calendar time, temperature, and relative humidity.
