# Supplementary material for: Fine Particulate Air Pollution and Hospital Utilization for Upper Respiratory Tract Infections in Beijing, China
Source: Int J Environ Res Public Health. 2019 Feb 13;16(4):533. doi: 10.3390/ijerph16040533 (PMC6406703; doi:10.3390/ijerph16040533)
Supplement: Supplementary file 1 [file ijerph-16-00533-s001.pdf]

Supplementary File

Table S1. Percentage change with 95% CI in all three kinds of hospital utilizations for URTI related to a 10 μg/m^3 increase in fine particulate matter ($PM_{2.5}$) level at lag 3 days, by different degrees of freedom (*df*) for calendar time, temperature, and relative humidity.

Hospital Service	Variable	df	Percentage Change	95% CI	*p*-Value
Emergency room visits	Calendar time	10	−0.19	−0.43, 0.05	0.117
		12	−0.16	−0.38, 0.07	0.175
		14a	−0.16	−0.39, 0.07	0.166
		16	−0.11	−0.33, 0.10	0.303
		18	−0.12	−0.33, 0.10	0.285
	Temperature	2	−0.16	−0.39, 0.07	0.166
		3a	−0.16	−0.39, 0.07	0.166
		4	−0.16	−0.39, 0.06	0.158
		5	−0.16	−0.39, 0.06	0.163
		6	−0.16	−0.39, 0.07	0.164
	Relative humidity	2	−0.16	−0.39, 0.07	0.166
		3a	−0.16	−0.39, 0.07	0.166
		4	−0.16	−0.39, 0.07	0.166
		5	−0.16	−0.39, 0.07	0.165
		6	−0.16	−0.39, 0.07	0.166
Hospital admissions	Calendar time	10	0.54	0.02, 1.06	0.043
		12	0.54	0.02, 1.06	0.043
		14a	0.59	0.06, 1.11	0.029
		16	0.60	0.07, 1.12	0.026
		18	0.61	0.08, 1.13	0.024
	Temperature	2	0.59	0.06, 1.11	0.029
		3a	0.59	0.06, 1.11	0.029
		4	0.59	0.06, 1.11	0.029
		5	0.59	0.06, 1.11	0.029
		6	0.59	0.06, 1.11	0.029
	Relative humidity	2	0.59	0.06, 1.11	0.029
		3a	0.59	0.06, 1.11	0.029
		4	0.59	0.06, 1.11	0.029
		5	0.59	0.06, 1.11	0.029
		6	0.59	0.06, 1.11	0.029
Outpatient visits	Calendar time	10	0.01	−0.28, 0.30	0.937
		12	0.01	−0.28, 0.30	0.943
		14a	0.01	−0.27, 0.30	0.918
		16	0.06	−0.22, 0.34	0.680
		18	0.04	−0.24, 0.31	0.792
	Temperature	2	0.01	−0.27, 0.30	0.918
		3a	0.01	−0.27, 0.30	0.918
		4	0.01	−0.27, 0.30	0.918
		5	0.01	−0.27, 0.30	0.918
		6	0.01	−0.27, 0.30	0.918
	Relative humidity	2	0.01	−0.27, 0.30	0.918
		3a	0.01	−0.27, 0.30	0.918
		4	0.01	−0.27, 0.30	0.921
		5	0.01	−0.27, 0.30	0.923
		6	0.01	−0.27, 0.30	0.925
Total hospital visits	Calendar time	10	0.01	−0.27, 0.29	0.941
		12	0.01	−0.27, 0.29	0.950
		14a	0.01	−0.26, 0.28	0.919
		16	0.06	−0.21, 0.32	0.665
		18	0.04	−0.22, 0.30	0.783

Temperature	2	0.01	−0.26, 0.28	0.919
	3a	0.01	−0.26, 0.28	0.919
	4	0.01	−0.26, 0.28	0.919
	5	0.01	−0.26, 0.28	0.919
	6	0.01	−0.26, 0.28	0.919
Relative humidity	2	0.01	−0.26, 0.28	0.919
	3a	0.01	−0.26, 0.28	0.919
	4	0.01	−0.26, 0.28	0.929
	5	0.01	−0.26, 0.28	0.930
	6	0.01	−0.26, 0.28	0.928

a: The *df* value used in this study.

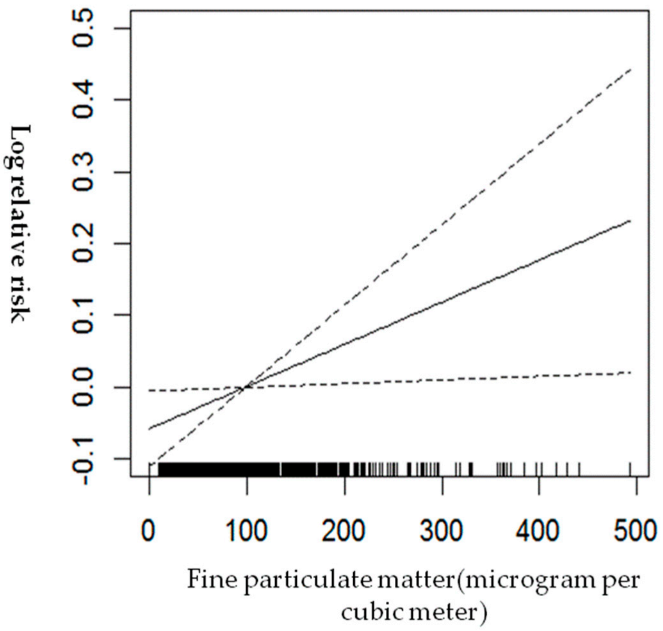

Figure S1. The exposure–response curve of fine particulate matter ($PM_{2.5}$) concentrations at lag 3 days and hospital admissions for URTI between 1 October 2010 and 30 September 2012 in Beijing, China. Note: The x-axis is the $PM_{2.5}$ concentrations (mg/m^3) at lag 3 days. The y-axis is the predicted log (relative risk (RR)). The curve, after adjusting for temperature, relative humidity, day of week, public holiday, and calendar time, is shown by the solid line, and the dotted lines represent the 95% CI.
